# 

**Published:** 1872-10

**Authors:** 


THE EDITORIAL AND PROPRIETARY DEPARTMENTS of this Journal are entirely *distinct.* Communications connected with the Editorial Department will be directed to the EDITORS, and those on business will be directed to the ATLANTA MEDICAL AND SURGICAL JOURNAL, care of "Plantation Publishing Company."

☞ *The Editors are not responsible for the views of Contributors.*

